# A Remark on Torsors under Affine Group Schemes

**DOI:** 10.1007/s00031-022-09767-z

**Published:** 2022-08-26

**Authors:** Michael Wibmer

**Affiliations:** Institute of Analysis and Number Therory, Graz University of Technology, Kopernikusgasse 24, 8010 Graz, Austria

**Keywords:** 14L15, 14L30, 14M17

## Abstract

We present an elementary proof of the fact that every torsor under an affine group scheme over an algebraically closed field is trivial. This is related to the uniqueness of fibre functors on neutral Tannakian categories.

## Introduction

Clearly, every torsor under an affine group scheme of finite type over an algebraically closed field is trivial. However, it is not clear if this also holds without the finite type assumption. This question was raised in [3], where some partial positive results were obtained: When the affine group scheme *G* is written as a projective limit of affine group schemes of finite type indexed by a set *I*, then all torsors under *G* are trivial if either *I* is countable or the cardinality of the algebraically closed base field is strictly greater than the cardinality of *I*. The main result discussed in this short note is the following:

### **Theorem 1.1**

Let *G* be an affine group scheme over an algebraically closed field *k* and let *X* be a torsor under *G*. Then, *X* is trivial, i.e. *X*(*k*)≠*∅*.

An equivalent reformulation of Theorem 1.1 in terms of Tannakian categories is:

### **Theorem 1.2**

Two neutral fibre functors on a neutral Tannakian category over an algebraically closed field are isomorphic.

We note that a different proof of Theorem 1.2 is outlined in [2], where the neutral Tannakian category is considered as a union of appropriate subcategories and a gluing principle for fibre functors is used (see [1, Sec. 6.4]).

In this note, we present an elementary proof of Theorem 1.1 that relies on a general principle guaranteeing the non-emptiness of a projective limit. We also show that (over an arbitrary base field) every torsor under *G* can be written as a projective limit of affine *G*-spaces that are torsors under finite type quotient groups of *G* (Proposition 2.3).

The author is grateful to Ch. Deninger and the anonymous referee for helpful comments.

## Proofs

We begin by fixing our notation. Throughout *k* is a field, the base field. All schemes (including group schemes), products, tensor products and morphisms are assumed to be over *k* unless the contrary is indicated.

We will often identify a scheme *X* with its functor of points $R\rightsquigarrow X(R)$ from the category of *k*-algebras to the category of sets. For an affine scheme *X*, we denote with *k*[*X*] its *k*-algebra of global sections.

Let *G* be an affine group scheme. By a *closed subgroup* of *G*, we mean a closed subgroup scheme of *G*. A *G-space* is a scheme *X* together with a *G*-action (from the right) *X* × *G* → *X*, (*x*,*g*)↦*x*.*g*. A morphism of *G*-spaces is a *G*-equivariant morphism of schemes. A *torsor under* *G* is a *G*-space *X* such that *X* × *G* → *X* × *X*, (*x*,*g*)↦(*x*,*x*.*g*), is an isomorphism. For an affine *G*-space *X*, the *centralizerC*_*G*_(*X*) is defined by

$$ C_{G}(X)(R):=\{g\in G(R)|\ x.g=x \ \forall \ x\in X(R^{\prime}) \text{ with } R^{\prime} \text{ an } R\text{-algebra}\}. $$

Then, *C*_*G*_(*X*) is a normal closed subgroup of *G* [4, Ch. II, Thm. 3.6 c] and *X* is a *G*/*C*_*G*_(*X*)-space. Recall that a faithfully flat morphism *G* → *H* of affine group schemes is called a *quotient morphism*; this is equivalent to the dual morphism *k*[*H*] → *k*[*G*] of Hopf algebras being injective (see [11, Sec. 14]).

Before entering into the proof of Theorem 1.1, let us establish the equivalence of Theorems 1.1 and 1.2. Assume that *ω*_1_ and *ω*_2_ are two neutral fibre functors on a neutral Tannakian category over an algebraically closed field *k*. Then, $G:=\underline {\text {Aut}}^{\otimes }(\omega _{1})$ is an affine group scheme and $\underline {\text {Isom}}(\omega _{1},\omega _{2})$ is a torsor under *G* [5, Thm. 3.2 (a)]. By Theorem 1.1, the torsor $\underline {\text {Isom}}(\omega _{1},\omega _{2})$ has a *k*-point, i.e. *ω*_1_ and *ω*_2_ are isomorphic.

Conversely, let *G* be an affine group scheme over an algebraically closed field *k* and let *X* be a torsor under *G*. Let *ω* be the forgetful functor on the neutral Tannakian category Rep(*G*) of finite dimensional *k*-linear representations of *G*. By [5, Thm. 3.2 (b)], the functor $\eta \rightsquigarrow \underline {\text {Isom}}^{\otimes }(\omega ,\eta )$ is an equivalence of categories between the category of neutral fibre functors on Rep(*G*) and the category of torsors under *G*. In particular, $X\simeq \underline {\text {Isom}}^{\otimes }(\omega ,\eta )$ for some neutral fibre functor *η* on Rep(*G*). By Theorem 1.2, the scheme $\underline {\text {Isom}}^{\otimes }(\omega ,\eta )$ has a *k*-point and therefore so does *X*. Thus, Theorems 1.1 and 1.2 are equivalent.

Let us now sketch the proof of Theorem 1.1. We are given a torsor *X* under an affine group scheme *G* and we would like to show that *X*(*k*)≠*∅*. We can write *X* as a projective limit $X=\varprojlim X_{i}$ of *G*-spaces *X*_*i*_ of finite type. So $X(k)=\varprojlim X_{i}(k)$. As we assume *k* to be algebraically closed, the *X*_*i*_(*k*)’s are non-empty. However, a projective limit of non-empty sets may well be empty. A standard condition to guarantee the non-emptiness of a projective limit of sets is that the sets are compact Hausdorff topological spaces with continuous transition maps [8, Prop. 1.1.4]. Unfortunately, the *X*_*i*_(*k*)’s equipped with the Zariski topology are not Hausdorff and so another approach is needed. The following lemma (see [7, Prop. 2.7] or [9, Thm. 2.1]) provides a more refined criterion to show that a projective limit is non-empty.

### **Lemma 2.1**

Let *I* be a directed set and let ((*X*_*i*_)_*i*∈*I*_,(*φ*_*i*,*j*_)_*i*≤*j*_) be a projective system of topological spaces. If the *X*_*i*_’s are non-empty compact T1 spaces and the *φ*_*i*,*j*_’s are closed maps, then $\varprojlim X_{i}$ is non-empty.

Returning to the above discussion, the *X*_*i*_(*k*)’s are compact T1 spaces with respect to the Zariski topology. However, the transition maps need not be closed and so Lemma 2.1 cannot be applied directly. A different topology is needed. We first show that the *X*_*i*_’s can be chosen in such a way that *X*_*i*_ is a torsor under *G*/*C*_*G*_(*X*_*i*_). Using this property, we show that the subsets of *X*_*i*_(*k*) that are finite unions of orbits of the form *x*.*H*(*k*) with *x* ∈ *X*_*i*_(*k*) and *H* a closed subgroup of *G* are the closed subsets of a topology on *X*_*i*_(*k*), the *orbit topology*. With respect to the orbit topology, the *X*_*i*_(*k*)’s are compact T1 spaces and the transition maps are continuous and closed. Thus, Lemma 2.1 applied to the projective system of the *X*_*i*_(*k*)’s equipped with the orbit topology yields Theorem 1.1.

To make the above sketch precise, we will use the action of *G* on *k*[*X*]. Let *G* be an affine group scheme and *X* an affine *G*-space. The *G*-action *X* × *G* → *X* induces a functorial (left) action of *G* on *k*[*X*]. For every *k*-algebra *R*, the group *G*(*R*) acts on *k*[*G*] ⊗ *R* by *R*-algebra automorphisms. Identifying *k*[*X*] ⊗ *R* with the set of morphisms from *X*_*R*_ to ${\mathbb {A}^{1}_{R}}$, the action of *g* ∈ *G*(*R*) on *f* ∈ *k*[*X*] ⊗ *R* is given by *g*(*f*)(*x*) = *f*(*x*.*g*) for all $x\in X(R^{\prime })$ with $R^{\prime }$ an *R*-algebra. The invariant ring under this action is

$$ k[X]^{G}:=\{f\in k[X]|\ g(f\otimes 1)=f\otimes 1 \ \forall\ g\in G(R) \text{ and every } k\text{-algebra }R\}. $$

It is a *k*-subalgebra of *k*[*X*]. Note that for a normal closed subgroup *N* of an affine group scheme *G* acting via right-multiplication on *G*, we have *k*[*G*]^*N*^ = *k*[*G*/*N*]. See, e.g. [11, Sec. 16.3].

### **Lemma 2.2**

Let *X* × *G* → *X* and *Y* × *H* → *Y* be actions of affine group schemes on affine schemes. With respect to the diagonal action of *G* × *H* on *X* × *Y*, we have

$$ k[X\times Y]^{G\times H}=k[X]^{G}\otimes k[Y]^{H}. $$

### *Proof*

Note that for a *k*-algebra *R* and *g* ∈ *G*(*R*), *h* ∈ *H*(*R*), the action of (*g*,*h*) is given by

$$ (g,h)\colon k[X\times Y]\otimes R=(k[X]\otimes R)\otimes_{R} (k[Y]\otimes R)\xrightarrow{g\otimes h} (k[X]\otimes R)\otimes_{R} (k[Y]\otimes R)=k[X\times Y]\otimes R. $$

So the inclusion $k[X]^{G}\otimes k[Y]^{H}\subseteq k[X\times Y]^{G\times H}$ is clear.

Conversely, assume that $\sum a_{i}\otimes b_{i}\in (k[X]\otimes k[Y])^{G\times H}$. We may assume that the *a*_*i*_’s are *k*-linearly independent. For every *k*-algebra *R* and *h* ∈ *H*(*R*), we have

$$ (1,h)\big(\sum a_{i}\otimes b_{i}\otimes 1\big)=\sum a_{i}\otimes h(b_{i}\otimes 1)=\sum a_{i}\otimes b_{i}\otimes 1\in k[X]\otimes k[Y]\otimes R $$

As the *a*_*i*_’s are *k*-linearly independent, we can conclude that *h*(*b*_*i*_ ⊗ 1) = *b*_*i*_ ⊗ 1, i.e. *b*_*i*_ ∈ *k*[*Y* ]^*H*^.

Now, assuming that the *b*_*i*_’s are *k*-linearly independent, a similar argument shows that the *a*_*i*_’s must lie in *k*[*X*]^*G*^. Thus, $\sum a_{i}\otimes b_{i}\in k[X]^{G}\otimes k[Y]^{H}$. □

It is well-known (see, e.g. [11, Sec. 3.3]) that every affine group scheme is a projective limit of affine algebraic groups. The following proposition shows that a similar statement is true for torsors.

### **Proposition 2.3**

Let *G* be an affine group scheme and let *X* be an affine torsor under *G*. Then, *X* can be written as a projective limit $X=\varprojlim _{i\in I} X_{i}$ of affine *G*-spaces *X*_*i*_ of finite type such that every *X*_*i*_ is a torsor under *G*/*C*_*G*_(*X*_*i*_).

### *Proof*

If an abstract group $\mathcal {G}$ acts (from the right) on a set $\mathcal {X}$ such that $\mathcal {X}$ is a torsor under $\mathcal {G}$, then for every normal subgroup $\mathcal {N}$ of $\mathcal {G}$ the set $\mathcal {X}/\mathcal {N}$ of $\mathcal {N}$-orbits in $\mathcal {X}$ is a torsor under $\mathcal {G}/\mathcal {N}$ via the action $\mathcal {X}/\mathcal {N}\times \mathcal {G}/\mathcal {N}\to \mathcal {X}/\mathcal {N},\ (x.\mathcal {N},g\mathcal {N})\mapsto x.g.\mathcal {N}$. This is the idea for the construction of the *X*_*i*_’s. However, to avoid a discussion of the existence of *X*/*N* (as an affine scheme) in our context, we will mainly work with the invariant rings.

Let *N* be a normal closed subgroup of *G* such that *G*/*N* is algebraic (i.e. of finite type). Then, *N* acts (form the right) on *X* and on *G*. Let *ρ*: *k*[*X*] → *k*[*X*] ⊗ *k*[*G*] be the dual of the action *X* × *G* → *X*. We claim that *ρ* restricts to a morphism *k*[*X*]^*N*^ → *k*[*X*]^*N*^ ⊗ *k*[*G*]^*N*^.

We have a (right) action of *N* × *N* on *X* × *G* given by (*x*,*g*).(*n*_1_,*n*_2_) = (*x*.*n*_1_,*gn*_2_) for *x* ∈ *X*(*R*), *g* ∈ *G*(*R*), *n*_1_,*n*_2_ ∈ *N*(*R*) and *R* a *k*-algebra. According to Lemma 2.2, the invariants *k*[*X* × *G*]^*N*×*N*^ with respect to this action are equal to *k*[*X*]^*N*^ ⊗ *k*[*G*]^*N*^. It thus suffices to show that *ρ* maps an *N*-invariant *f* ∈ *k*[*X*] to an (*N* × *N*)-invariant, i.e. we have to show that *f*(*x*.*g*) = *f*(*x*.*n*_1_*gn*_2_) for *n*_1_,*n*_2_ ∈ *N*(*R*), $x\in X(R^{\prime })$, $g\in G(R^{\prime })$, where *R* is a *k*-algebra and $R^{\prime }$ an *R*/¯algebra. But as $g^{-1}n_{1}gn_{2}\in N(R^{\prime })$, we have *f*(*x*.*g*) = *f*(*x*.*g*.*g*^− 1^*n*_1_*gn*_2_) = *f*(*x*.*n*_1_*gn*_2_) by the *N*-invariance of *f*.

Thus, *ρ* restricts to a well-defined map *ρ*_*N*_: *k*[*X*]^*N*^ → *k*[*X*]^*N*^ ⊗ *k*[*G*]^*N*^. Setting *X*_*N*_ := Spec(*k*[*X*]^*N*^), we thus have an action *X*_*N*_ × *G*/*N* → *X*_*N*_ of *G*/*N* on *X*_*N*_. We claim that *X*_*N*_ is a torsor under *G*/*N*.

The dual *ψ*: *k*[*X*] ⊗ *k*[*X*] → *k*[*X*] ⊗ *k*[*G*] of the isomorphism *X* × *G* → *X* × *X*, (*x*,*g*)↦(*x*,*x*.*g*), is an isomorphism. Therefore, the dual *ψ*_*N*_: *k*[*X*]^*N*^ ⊗ *k*[*X*]^*N*^ → *k*[*X*]^*N*^ ⊗ *k*[*G*]^*N*^ of *X*_*N*_ × *G*/*N* → *X*_*N*_ × *X*_*N*_, (*x*,*g*)↦(*x*,*x*.*g*), is at least injective.

To see that *ψ*_*N*_ is surjective, we consider the (*N* × *N*)-invariants on both sides of the isomorphism *ψ* (Lemma 2.2). Note, however, that *ψ* is not (*N* × *N*)-equivariant. Nevertheless, to show that *ψ*_*N*_ is surjective, it suffices to show that *ψ*(*f*) ∈ (*k*[*X*] ⊗ *k*[*G*])^*N*×*N*^ for *f* ∈ *k*[*X*] ⊗ *k*[*X*] implies *f* ∈ (*k*[*X*] ⊗ *k*[*X*])^*N*×*N*^. But *ψ*(*f*) ∈ (*k*[*X*] ⊗ *k*[*G*])^*N*×*N*^ means that

1 $$ f(x,x.g)=\psi(f)(x,g)=\psi(f)(x.n_{1},g.n_{2})=f(x.n_{1},x.n_{1}gn_{2}) $$

for *n*_1_,*n*_2_ ∈ *N*(*R*), $x\in X(R^{\prime })$, $g\in G(R^{\prime })$, *R* a *k*-algebra and $R^{\prime }$ an *R*-algebra.

Given a *k*-algebra $\widetilde {R}$, elements $\widetilde {n}_{1}, \widetilde {n}_{2}\in N(\widetilde {R})$ and $x_{1},x_{2}\in X(\widetilde {R}^{\prime })$ with $\widetilde {R}^{\prime }$ an $\widetilde {R}$-algebra, we can, using that *X* is a torsor under *G*, write $x_{2}=x_{1}.\widetilde {g}$ for a unique $\widetilde {g}\in G(\widetilde {R}^{\prime })$. Then, using Eq. 1 with $R=R^{\prime }=\widetilde {R}^{\prime }$, *x* = *x*_1_, $g=\widetilde {g}$, $n_{1}=\widetilde {n}_{1}$, $n_{2}=\widetilde {g}^{-1}\widetilde {n}_{1}^{-1}\widetilde {g}\widetilde {n}_{2}\in N(\widetilde {R}^{\prime })$, we have

$$ f(x_{1},x_{2})=f(x_{1},x_{1}.\widetilde{g})=f(x_{1}.\widetilde{n}_{1}, x_{1}.\widetilde{n}_{1}\widetilde{g}\widetilde{g}^{-1}\widetilde{n}_{1}^{-1}\widetilde{g}\widetilde{n}_{2})=f(x_{1}.\widetilde{n}_{1},x_{1}. \widetilde{g}\widetilde{n}_{2})=f(x_{1}.\widetilde{n}_{1},x_{2}.\widetilde{n}_{2}). $$

Thus, *f* ∈ (*k*[*X*] ⊗ *k*[*X*])^*N*×*N*^ = *k*[*X*]^*N*^ ⊗ *k*[*X*]^*N*^ as desired and we conclude that *X*_*N*_ is a torsor under *G*/*N*. Because *G*/*N* is algebraic, also *X*_*N*_ has to be of finite type. Indeed, a *K*-point of *X*_*N*_ in some field extension *K* of *k* yields an isomorphism of *K*-algebras between *k*[*G*/*N*] ⊗ *K* and *k*[*X*_*N*_] ⊗ *K*. As *k*[*G*/*N*] is a finitely generated *k*-algebra, we see that *k*[*G*/*N*] ⊗ *K* and therefore also *k*[*X*_*N*_] ⊗ *K* are finitely generated *K*-algebras, which implies that *k*[*X*_*N*_] is a finitely generated *k*-algebra.

We next show that *k*[*X*] is the directed union of the *k*[*X*]^*N*^’s. Because each *k*[*X*]^*N*^ is a finitely generated *k*-algebra, it suffices to show that every finite subset *F* of *k*[*X*] is contained in some *k*[*X*]^*N*^.

Note that *ρ*: *k*[*X*] → *k*[*X*] ⊗ *k*[*G*] defines the structure of a (right) comodule on *k*[*X*]. According to [11, Thm. 3.3], every comodule is the directed union of its finite dimensional (as *k*/¯vector space) comodules. So *F* is contained in a finite dimension *k*-subspace *V* of *k*[*X*] such that $\rho (V)\subseteq V\otimes k[G]$. Let *A* be the *k*-subalgebra of *k*[*X*] generated by *V*. Then, *A* is finitely generated and $\rho (A)\subseteq A\otimes k[G]$. In fact, as *A* is finitely generated, there exists a finitely generated *k*-subalgebra *B* of *k*[*G*] such that $\rho (A)\subseteq A\otimes B$. According to [11, Sec. 3.3], every Hopf algebra is the directed union of Hopf subalgebras that are finitely generated as *k*-algebras. Thus, *B* is contained in some Hopf subalgebra $B^{\prime }$ of *k*[*G*] that is finitely generated as a *k*-algebra. Every Hopf subalgebra of *k*[*G*] is of the form *k*[*G*/*N*] = *k*[*G*]^*N*^ for a normal closed subgroup *N* of *G* ([11, Sec. 15 and 16] or [10, Thm. 4.3]). Thus, $B^{\prime }=k[G]^{N}$ for a normal closed subgroup *N* of *G* with *G*/*N* algebraic. Moreover, $F\subseteq A$ and $\rho (A)\subseteq A\otimes k[G]^{N}$. It thus suffices to show that $A\subseteq k[X]^{N}$. For $f\in A\subseteq k[X]$, we have *ρ*(*f*) ∈ *k*[*X*] ⊗ *k*[*G*]^*N*^. This means that for a *k*-algebra *R*, an *R*-algebra $R^{\prime }$, *n* ∈ *N*(*R*), $g\in G(R^{\prime })$ and $x\in X(R^{\prime })$, we have *f*(*x*.*g*) = *f*(*x*.*gn*). Choosing *g* = 1, we see that *f* ∈ *k*[*X*]^*N*^. So $A\subseteq k[X]^{N}$ as desired.

Note that $k[X]^{N}\subseteq k[X]^{N^{\prime }}$ if $N^{\prime }\subseteq N$. As *k*[*X*] is the directed union of the *k*[*X*]^*N*^’s, we see that $X=\varprojlim X_{N}$, where the projective limit is taken over the set of all closed normal subgroups *N* of *G* such that *G*/*N* is algebraic. This index set is a directed set with respect to the partial order defined by $N\leq N^{\prime }$ if $N^{\prime }\subseteq N$.

To finish the proof, it remains to verify that *C*_*G*_(*X*_*N*_) = *N*. As the action of *G* on *X*_*N*_ factors through *G*/*N*, surely $N\subseteq C_{G}(X_{N})$. Conversely, if *R* is a *k*-algebra and *g* ∈ *C*_*G*_(*X*_*N*_)(*R*), then the image $\overline {g}$ of *g* in (*G*/*N*)(*R*) acts trivially on $X_{N}(R^{\prime })$ for every *R*-algebra $R^{\prime }$. As *X*_*N*_ is a torsor under *G*/*N*, we must have $\overline {g}=1$, i.e. *g* ∈ *N*(*R*). Thus, $C_{G}(X_{N})\subseteq N$ and consequently *C*_*G*_(*X*_*N*_) = *N*. □

The following lemma introduces the orbit topology on *X*(*k*), where *X* is a *G*-space such that *X* is a torsor under *G*/*C*_*G*_(*X*). This topology is similar to the topology on the *k*-points of an affine algebraic group discussed before [7, Prop. 2.8].

### **Lemma 2.4**

Assume that *k* is algebraically closed and *G* is an affine group scheme.

(i) Let *X* be an affine *G*-space of finite type such that *X* is a torsor under *G*/*C*_*G*_(*X*). Then, the subsets of *X*(*k*) that are finite unions of orbits of the form *x*.*H*(*k*) with *x* ∈ *X*(*k*) and *H* a closed subgroup of *G* are the closed subsets for a topology on *X*(*k*). With respect to this topology, to be called the *orbit topology*, *X*(*k*) is a compact T1 space.
(ii) Let *ϕ*: *X*_2_ → *X*_1_ be a morphism of affine *G*-spaces of finite type such that *X*_*i*_ is a torsor under *G*/*C*_*G*_(*X*_*i*_) (*i* = 1,2). Then, the map *ϕ*_*k*_: *X*_2_(*k*) → *X*_1_(*k*) is continuous and closed with respect to the orbit topologies.

### *Proof*

For (i), we first argue that *X*(*k*) is closed with respect to the orbit topology. As *X* is of finite type and *k* is algebraically closed, there exists an *x* ∈ *X*(*k*). Because the map *G*(*k*) → (*G*/*C*_*G*_(*X*))(*k*) is surjective [4, Ch. III, §3, Cor. 7.6] and *X* is a torsor under *G*/*C*_*G*_(*X*), we see that *X*(*k*) = *x*.*G*(*k*). Thus, *X*(*k*) is closed with respect to the orbit topology.

We next show that an orbit of the form *x*.*H*(*k*) with *x* ∈ *X*(*k*) and *H* a closed subgroup of *G* is a closed subset of *X*(*k*) with respect to the Zariski topology. Set $G^{\prime }:=G/C_{G}(X)$ and let $H^{\prime }$ denote the image of *H* in $G^{\prime }$. Then, $H\to H^{\prime }$ is a quotient morphism and by [4, Ch. III, §3, Cor. 7.6] the map $H(k)\to H^{\prime }(k)$ is surjective. Thus, $x.H(k)=x.H^{\prime }(k)$. As *X* is a torsor under $G^{\prime }$, the morphism $G^{\prime }\to X,\ g^{\prime }\mapsto x.g^{\prime }$ is an isomorphism. In particular, $G^{\prime }(k)\to X(k)$ is a homeomorphism mapping the closed subset $H^{\prime }(k)$ to the closed subset $x.H^{\prime }(k)$. So *x*.*H*(*k*) is closed with respect to the Zariski topology and so is every finite union of such orbits.

As *X* is of finite type, every descending chain of Zariski closed subsets of *X* is finite. Thus, an arbitrary intersection of finite unions of orbits is in fact a finite intersection of finite unions of orbits. Therefore, to show that an arbitrary intersection of finite unions of orbits is itself a finite union of orbits, it suffices to show that the intersection of two orbits is again an orbit. So let *H*_1_,*H*_2_ be closed subgroups of *G* and *x*_1_,*x*_2_ ∈ *X*(*k*). If (*x*_1_.*H*_1_(*k*)) ∩ (*x*_2_.*H*_2_(*k*)) is non-empty, then there exists an *x* ∈ *X*(*k*) such that *x*_1_*H*_1_(*k*) = *xH*_1_(*k*) and *x*_2_*H*_2_(*k*) = *xH*_2_(*k*). Moreover, as noted above, we have $xH_{1}(k)=xH_{1}^{\prime }(k)$ and $xH_{2}^{\prime }(k)$ with $H_{i}^{\prime }$ the image of *H*_*i*_ in $G^{\prime }$. Then,

$$ (x_{1}.H_{1}(k))\cap (x_{2}.H_{2}(k))=(x.H^{\prime}_{1}(k))\cap (x.H^{\prime}_{2}(k)=x.(H^{\prime}_{1}(k)\cap H^{\prime}_{2}(k))=x.(H_{1}^{\prime}\cap H_{2}^{\prime})(k), $$

where the second equality uses that $G^{\prime }(k)$ acts freely on *X*(*k*). Thus, if *H* ≤ *G* denotes the inverse image of $H_{1}^{\prime }\cap H_{2}^{\prime }\leq G^{\prime }$ under the quotient morphism $G\to G^{\prime }$, then (*x*_1_.*H*_1_(*k*)) ∩ (*x*_2_.*H*_2_(*k*)) = *x*.*H*(*k*).

Therefore, the finite unions of orbits are indeed the closed sets of a topology on *X*(*k*). As noted above, a subset of *X*(*k*) that is closed with respect to the orbit topology is closed with respect to the Zariksi topology. In particular, every descending chain of closed subsets with respect to the orbit topology is finite. Hence, *X*(*k*) is compact with respect to the orbit topology. The points of *X*(*k*) are closed with respect to the orbit topology because they are the orbits of the trivial subgroup *H* = 1 of *G*. This concludes the proof of (i).

For (ii), we first show that *ϕ*_*k*_: *X*_2_(*k*) → *X*_1_(*k*) is surjective. Let *x*_1_ ∈ *X*_1_(*k*). The group *G*(*k*) acts transitively on *X*_1_(*k*) because *G*(*k*) → (*G*/*C*_*G*_(*X*_1_))(*k*) is surjective (again by [4, Ch. III, Cor. 7.6]) and *X*_1_(*k*) is a torsor under (*G*/*C*_*G*_(*X*_1_))(*k*). Thus, if *x*_2_ is an element of *X*_2_(*k*), there exists a *g* ∈ *G*(*k*) such that *x*_1_ = *ϕ*_*k*_(*x*_2_).*g* = *ϕ*_*k*_(*x*_2_.*g*). Hence, *ϕ*_*k*_ is surjective.

To show that *ϕ*_*k*_ is continuous with respect to the orbit topologies, it suffices to show that the inverse image of an orbit is on orbit. So let *H* be a closed subgroup of *G* and *x*_1_ ∈ *X*_1_(*k*). We would like to show that $\phi _{k}^{-1}(x_{1}.H(k))$ is an orbit. As noted in the proof of (i), we have $x_{1}.H(k)=x_{1}.H^{\prime }(k)$, where $H^{\prime }$ denotes the image of *H* in *G*/*C*_*G*_(*X*_1_). In other words, we may assume that *C*_*G*_(*X*_1_) ≤ *H*. As *ϕ*_*k*_ is surjective, there exists an *x*_2_ ∈ *X*_2_(*k*) such that *ϕ*_*k*_(*x*_2_) = *x*_1_. We claim that $\phi _{k}^{-1}(x_{1}.H(k))=x_{2}.H(k)$. Clearly, $x_{2}.H(k)\subseteq \phi _{k}^{-1}(x_{1}.H(k))$. For the reverse inclusion, let $x_{2}^{\prime }\in \phi _{k}^{-1}(x_{1}.H(k))$. As *G*(*k*) acts transitively on *X*_2_(*k*), there exists a *g* ∈ *G*(*k*) such that $x^{\prime }_{2}=x_{2}.g$. Then,

$$ x_{1}.g=\phi_{k}(x_{2}).g=\phi_{k}(x_{2}.g)=\phi_{k}(x^{\prime}_{2})\in x_{1}.H(k). $$

Hence, there exists an *h* ∈ *H*(*k*) such that *x*_1_.*g* = *x*_1_.*h*. As *X*_1_(*k*) is a torsor under (*G*/*C*_*G*_(*X*_1_))(*k*), we have *gh*^− 1^ ∈ *C*_*G*_(*X*_1_)(*k*). But *C*_*G*_(*X*_1_) ≤ *H* and so *g* ∈ *H*(*k*). Thus, $x_{2}^{\prime }=x_{2}.g\in x_{2}.H(k)$. Therefore, $\phi _{k}^{-1}(x_{1}.H(k))=x_{2}.H(k)$ and *ϕ*_*k*_ is continuous with respect to the orbit topologies.

To see that *ϕ*_*k*_ is closed with respect to the orbit topologies, it suffices to see that *ϕ*_*k*_ preserves orbits. But this follows immediately from the *G*(*k*)-equivariance of *ϕ*_*k*_. □

We are now prepared to prove the main result.

### *Proof Proof of Theorem 1.1*

We first note that *X* is an affine scheme. Indeed, *X* and *G* become isomorphic over some field extension *K* of *k*. So *X*_*K*_ is an affine scheme. By faithfully flat descent, the morphism *X* →Spec(*k*) is affine (see [6, Exposé VIII, Cor. 5.6]), so *X* is an affine scheme.

By Proposition 2.3, we may write *X* as a projective limit $X=\varprojlim _{i\in I} X_{i}$ of affine *G*-spaces *X*_*i*_ of finite type such that each *X*_*i*_ is a torsor under *G*/*C*_*G*_(*X*_*i*_). In particular, $X(k)=\varprojlim _{i\in I} X_{i}(k)$.

By Lemma 2.4, each *X*_*i*_(*k*) is a compact T1 space with respect to the orbit topology and the transition maps *X*_*j*_(*k*) → *X*_*i*_(*k*) (*j* ≥ *i*) are continuous and closed with respect to the orbit topologies. Thus, Lemma 2.1 applied to the projective system of the *X*_*i*_(*k*)’s equipped with the orbit topology shows that *X*(*k*) is non-empty. □
